# Knowledge and Attitudes Regarding the Inappropriate Use of Proton Pump Inhibitors Among Students of Umm Al-Qura University in Saudi Arabia: A Cross-Sectional Study

**DOI:** 10.7759/cureus.71282

**Published:** 2024-10-11

**Authors:** Abdulfattah Y Alhazmi, Asayel N Alamri, Taif A Alayyafi, Lamah K Allehaibi, Mohammed Aldurdunji

**Affiliations:** 1 Pharmaceutical Practices Department, College of Pharmacy, Umm Al-Qura University, Makkah, SAU; 2 Pharmacology Department, College of Pharmacy, Umm Al-Qura University, Makkah, SAU

**Keywords:** gastrointestinal, inappropriate use, knowledge attitude, proton pump inhibitors (ppi), survey

## Abstract

Background

Proton pump inhibitors (PPIs) are commonly prescribed to manage various upper gastrointestinal conditions such as dyspepsia, gastroesophageal reflux disease (GERD), and peptic ulcer disease. However, concerns are increasing regarding their excessive and inappropriate use, particularly in cases where there is no clear medical indication. Inappropriate use can lead to unnecessary side effects and complications, emphasizing the need for better awareness of appropriate PPI use.

Objectives

This study aimed to assess the knowledge and attitudes of Umm Al-Qura University (UQU) students in Makkah, Saudi Arabia, regarding the inappropriate use of PPIs, highlighting the necessity of targeted educational interventions to improve awareness and promote safe PPI practices.

Methods

A cross-sectional online survey was conducted among 534 UQU students, aged 18-30, from both health-related and non-health-related majors. Participants were selected through convenience sampling, with the questionnaire distributed via Google Forms across various channels. Data were prepared using Microsoft Excel and analyzed with IBM SPSS Statistics for Windows, V. 25.0 (IBM Corp., Armonk, NY, USA).

Results

The survey revealed a significant lack of awareness about the potential side effects of PPIs, with 48.1% of participants (n=247) exhibiting low awareness, 30.9% (n=165) demonstrating high awareness, and 21% (n=112) showing moderate understanding. A chi-squared test confirmed that this awareness distribution significantly differed from what was expected, χ²(2, n=534)=60.48, p<0.001. In terms of behavior, 70.4% of participants adhered to good practices regarding PPI use, following appropriate guidelines such as using PPIs only when prescribed, following the correct dosage, and avoiding self-medication. Additionally, 21.3% (n=114) displayed moderate behavior, and 8.2% demonstrated poor behavior by engaging in practices such as using PPIs without medical supervision or not adhering to the recommended dosage and duration. A chi-squared test indicated significant differences in the behavior distribution, χ²(2, n=534)=344.14, p<0.001.

Conclusion

While most respondents displayed good behavior regarding PPI usage, there remains a significant gap in knowledge and attitudes. Addressing these gaps through targeted educational interventions, such as workshops, e-learning modules, and awareness campaigns, could promote the safe and effective use of PPIs among students in Saudi Arabia. The next steps would involve implementing these programs in collaboration with healthcare professionals and university departments, with effectiveness measured through follow-up surveys, behavior assessments, and analysis of PPI usage trends over time.

## Introduction

Proton pump inhibitors (PPIs) are medications that receive extensive prescriptions that belong to the class of drugs known as gastric acid inhibitors used for treating and preventing gastrointestinal problems [1]. They remain the preferred choice for medical professionals in managing upper gastrointestinal issues, including dyspepsia, gastroesophageal reflux disease (GERD), peptic ulcer diseases, and their associated complications [2]. Physicians often prescribe PPI therapy without a valid indication, such as when they want to prevent ulcers in patients who do not have risk factors (e.g., those who are only receiving steroid therapy) and excessively treat functional dyspepsia [3]. Besides, a study conducted in Saudi Arabia in 2019 found that all community pharmacists regularly recommend the use of PPIs to their patients, often regardless of whether there is a genuine medical need for the medication [4]. Inappropriate PPI use is associated with iatrogenic diseases, interactions with other drugs and nutrients, and increased healthcare costs [5]. Excessive and irrational use of PPIs can adversely affect therapeutic outcomes despite their proven effectiveness, tolerability, and safety [6]. Numerous studies have highlighted instances where PPI usage surpasses the reported cases of gastrointestinal issues [7]. The phenomenon of excessive dependence on PPIs has been well-documented in scientific literature from various regions worldwide [8].

Considering recent apprehensions surrounding the extended safety of PPIs, it is noteworthy that the balance between risks and benefits strongly supports their judicious utilization in patients with legitimate medical reasons for their prescription [9]. As with all pharmaceutical agents, PPIs should be administered at a minimally effective dosage and solely for the medically necessary duration [9]. Nonetheless, PPIs will likely be deemed indispensable continuously for specific officially sanctioned uses [9].

Like other medications, PPIs are not without potential side effects, particularly when misused [10]. They might be at an increased risk of hip fracture, lacking essential nutrients, or even the onset of *Clostridium difficile* infection [10]. Prolonged, unnecessary usage is also associated with adverse health outcomes such as vitamin B12 deficiency dementia, pneumonia, gastric cancer, and chronic kidney disease [11,12]. Therefore, the regulatory measures governing the use of PPIs follow guidance from the US Food and Drug Administration [1]. However, there remains a critical gap in the literature regarding the knowledge, attitudes, and behaviors of university students, particularly comparing those from healthcare and non-healthcare backgrounds, in relation to the inappropriate use of PPIs. To our knowledge, no previous study has specifically addressed this issue within a university student population in Saudi Arabia.

Objectives

Primary Objectives

This study aims to assess the attitudes of students at Umm Al-Qura University (UQU) towards the inappropriate use of PPIs. Additionally, it seeks to evaluate the behavior of students in relation to the potential risks associated with improper PPI usage.

Secondary Objectives

The secondary objectives of this study are to determine the prevalence of PPI usage among UQU students and identify the factors influencing their use. Additionally, the study aims to explore the sources of information that students rely on when using PPIs and assess the credibility of these sources. Finally, it seeks to compare the knowledge and attitudes regarding PPI use between healthcare and non-healthcare students at UQU in Makkah, Saudi Arabia.

## Materials and methods

Study design

The current study is a cross-sectional study conducted online to evaluate the knowledge and attitudes regarding the inappropriate utilization of PPIs among 534 students enrolled at UQU in Makkah, Saudi Arabia (2023-2024).

Study population

All students at UQU will be our target population (2023-2024).

Inclusion criteria

The inclusion criteria for this study are as follows: students enrolled in both healthcare and non-healthcare bachelor and bridging courses, aged between 18 and 30 years, and participants of both male and female genders. By excluding students in higher studies, such as those in master's and PhD programs, we ensure that the findings remain relevant to the undergraduate population. This focus is essential for designing targeted educational interventions.

Exclusion criteria

The exclusion criteria for this study include students who refused to participate and those who were not enrolled at UQU during the 2023-2024 academic year.

Study procedure

The study utilized an online questionnaire (see Appendices) to collect data from UQU students. The participants for our survey were selected using a convenience sampling approach. The questionnaire was distributed through various channels, including university email lists, social media platforms, and student forums. A link to the electronic questionnaire was shared widely to reach a broad audience within the university. To encourage participation, reminders were sent out periodically. After completing the questionnaire, data were cleaned and prepared for analysis using Microsoft Excel and analyzed using IBM SPSS Statistics for Windows, V. 25.0 (IBM Corp., Armonk, NY, USA).

Data collection management

Data collection was conducted using an electronic questionnaire available in both English and Arabic, distributed to the target population. The questionnaire used in this study was adopted from a validated and published study [1], after obtaining a verbal permission from the corresponding and primary author, Prof. Asdaq. Cronbach's alpha for internal consistency was 0.78, and consequently, the reliability of the survey was affirmed. This questionnaire included several key components: consent forms, demographic data, an assessment of participants' knowledge about PPIs, an evaluation of their attitudes towards PPI use, and specific questions directed at students who were currently using PPIs.

Sample size determination

The total number of participants was 534 students. The minimum recommended sample size of this survey was 383, with 95% confidence intervals. The sample size calculations utilized the online sample size calculator by Raosoft® (Raosoft Inc., Seattle, WA, USA).

Statistical analysis

The collected data were inputted into the SPSS statistical software and subjected to analysis. Utilizing the chi-squared test, an examination of the data was carried out. Descriptive analysis was employed to establish correlations between dependent variables (knowledge, attitudes) and sociodemographic characteristics. Significance was determined with a p-value less than 0.05. Multinomial regression analysis was conducted to identify the risk coefficient. Correlation statistics were applied to assess the relationship between knowledge and attitudes within each domain. The entire statistical analysis was performed using IBM SPSS Statistics for Windows, V. 25.0.

Ethical part and confidentiality

This study was approved by the Umm Al-Qura University Institutional Research Board (approval number: QTMB151223). Study activities were begun once permission was obtained. Survey responses were collected anonymously. We have not collected any identifying information from participants and private information, and all answers had their confidentiality maintained.

## Results

The number of participants in this study was 534 UQU students. Table 1 provides a detailed breakdown of the participants based on various parameters such as age, gender, faculty in the medical field, medical field specialization, and university year. It is evident from the data that the majority of participants fell within the age range of 18-30 years, comprising 78.8% of the total sample (n=421). In terms of gender distribution, females accounted for 83% of the participants (n=443), while males made up 17%. Furthermore, the data indicates that nearly half of the participants (49.1%) were affiliated with the faculty of the medical field, with a relatively equal split between those in the medical field (Pharmacy, Medicine, Dentistry, Nursing) and others. When considering the distribution across university years, the data shows a fairly even distribution among different academic levels, with the highest representation in the fourth year (21%) followed closely by the first year (21.3%). This comprehensive overview of the participants' sociodemographic characteristics provides valuable insights into the demographics of the study population, which can inform future research and interventions aimed at addressing the inappropriate use of PPIs among students.

**Table 1:** Sociodemographic characteristics of the participants (n=534)

Variable		N	%
Age (in years)	18-30	421	78.8
	31-40	74	13.9
	41-50	39	7.3
Gender	Female	443	83
	Male	91	17
Faculty of the medical field	Yes	262	49.1
	No	272	50.9
Medical field	Pharmacy	95	17.8
	Medicine	102	19.1
	Dentistry	23	4.3
	Nursing	68	12.7
	Other	246	46
University year	First year	114	21.3
	Second year	81	15.2
	Third year	84	15.7
	Fourth year	112	21
	Fifth year	70	13.1
	Sixth year	73	13.7

In the survey, respondents were asked whether they were affiliated with a medical field college. The data showed a varied distribution across different medical disciplines. A total of 246 students (46%) were in fields outside of the traditional medical disciplines. Among those in medical-related fields, 95 students (17.8%) were studying Pharmacy, 102 students (19.1%) were in Medicine, 23 students (4.3%) were in Dentistry, and 68 students (12.7%) were studying Nursing. This distribution highlights a diverse range of academic backgrounds within the medical- and health-related fields, which could influence responses and may be important for decisions or policies related to medical education or practice. The distribution of students across these fields can serve as a valuable reference point for further analysis and decision-making within the medical domain.

Table 2 showcases various aspects of PPI usage among the participants. It is evident from the data that a significant portion of the participants (23%) have used PPIs before, with a notable percentage (63.3%) indicating that they have never used them. Among those who have used PPIs, reasons for usage vary, with the majority citing not having used it before (52.85%) and conditions like peptic ulcer (15.17%) and GERD (11.8%). Interestingly, a substantial number of participants (60.67%) reported receiving recommendations to use PPIs from sources other than healthcare professionals. The duration of PPI usage varied, with a considerable proportion (52.2%) indicating they had not used it before. When it comes to the appropriate time to take PPIs, responses varied, with a significant number (56.7%) mentioning not having used it before. Completion of the course of PPIs also showed diverse responses, with a considerable percentage (56.2%) indicating "Other". Reasons for not completing the course included factors such as side effects, fear of side effects, and affordability issues. The number of students who answered "not used PPI before" varied across different parameters because the missing data in the analysis was handled case-wise rather than list-wise, resulting in differing respondent numbers for various parameters.

**Table 2:** Parameters related to knowledge about PPIs (n=534) PPI: proton pump inhibitor; GERD: gastroesophageal reflux disease

Parameter		N	%
Have you ever used PPI before?	Yes, by medical consultation	123	23
	Yes, without medical consultation	73	13.7
	I have never used it	338	63.3
If yes, the reason for using PPI?	Not used before	287	52.85
	Peptic ulcer	81	15.17
	GERD	63	11.8
	Heartburn	80	14.98
	H. pylori	23	4.31
	Esophagitis	16	3
	Gastritis/gastroenteritis	27	5.1
	Hiatal hernia	9	1.69
	Asthma	16	3
	Hoarseness	10	1.87
	Sore throat	15	2.81
	After bariatric surgery	10	1.87
Who recommended using PPI?	Other	324	60.67
	Physician	109	20.4
	Friend	63	11.8
	Pharmacist	70	13.11
Duration of using PPI if used	I have not used it before	279	52.2
	As needed	73	13.7
	Regularly more than four weeks	59	11
	Regularly less than four weeks	49	9.2
	Not regularly	30	5.6
	Used the medication but did not complete the recommended duration	44	8.2
Appropriate time to take PPI	I have not used it before	303	56.7
	Before meal	98	18.4
	After meal	84	15.7
	With meal	49	9.2
Completed the course, if used PPI	No	94	17.6
	Maybe	64	12
	Yes	76	14.2
	Other	300	56.2
If not, why? (n=509)	I have not used it before	284	53.18
	Due to side effects	38	7.16
	Fear of side effects	62	11.61
	Symptoms disappear, no longer used	49	9.18
	Advice from a friend or relative (inside the medical field)	5	0.94
	Advice from a friend or relative (outside the medical field)	18	3.37
	Missed	29	5.43
	Not affordable, not available	55	10.3

Table 3 reveals that a total of 534 participants were included in the study. The table provides a breakdown of responses indicating whether participants were aware of specific side effects of PPIs. Notably, a significant proportion of participants reported awareness of common side effects such as headache, bloating, nausea, diarrhea, constipation, and abdominal pain. However, there was a marked lack of awareness regarding more severe side effects, including deterioration of kidney function, osteoporosis, and gastric cancer, as indicated by the lower percentages of affirmative responses in these categories. Additionally, a substantial number of participants were unaware of potential side effects such as iron deficiency anemia, mineral deficiency, and B12 deficiency. This underscores the importance of educating individuals, particularly students, about the appropriate use of PPIs and the associated risks to promote safe and informed decision-making regarding their healthcare.

**Table 3:** Participants' knowledge and awareness about the side effects of PPI (n=534) Participants were asked whether they were aware that PPIs can cause the following side effects. Responses of "Yes" and "No" indicate their knowledge and awareness. PPI: proton pump inhibitor

Parameter	Yes	No
Headache	57 (10.7%)	477 (89.3%)
Bloating	55 (10.3%)	479 (89.7%)
Nausea	49 (9.2%)	485 (90.8%)
Diarrhea	46 (8.6%)	488 (91.4%)
Constipation	29 (5.4%)	505 (94.6%)
Deterioration of kidney function	15 (2.8%)	519 (97.2%)
Abdominal pain	16 (3%)	518 (97%)
Osteoporosis	15 (2.8%)	519 (97.2%)
Iron deficiency anemia	157 (29.4%)	377 (70.6%)
Mineral deficiency	8 (1.5%)	526 (98.5%)
Increased risk of gastroenteritis	4 (0.8%)	530 (99.2%)
Pneumonia	5 (0.95%)	529 (99.05%)
*Clostridium difficile* infection	8 (1.5%)	526 (98.5%)
B12 deficiency	12 (2.3%)	522 (97.7%)
Gastric cancer	4 (0.7%)	530 (99.3%)
Increased blood glucose level	1 (0.2%)	533 (99.8%)

Table 4 displays a breakdown of responses from 534 individuals on various parameters related to PPI overuse. The findings reveal that a significant portion of participants acknowledge the commonality of PPI overuse in Saudi Arabia, with a notable percentage expressing concerns about the reasons behind this phenomenon, attributing it to either doctors' or patients' abuse of PPI. Moreover, the data suggests a prevalent belief among participants that stress ulcer prophylaxis is a primary driver of PPI overuse. Additionally, there is a recognition of the potential consequences of PPI overuse, such as an increase in adverse drug reactions and medical costs. The study also highlights the perceived need for extensive education on the rational use of PPI among medical staff and the public, as well as the importance of enhancing the management of community pharmacies to address this issue effectively.

**Table 4:** Participants' attitudes regarding PPI use* (n=534) *Scoring systems were regulated based on the 5-point Likert scale with the scores being as follows: 5 for "completely agree", 4 for "almost agree", 3 for "indifferent", 2 for "almost disagree", and 1 for "completely disagree". The total score for the six questions was 30, and scoring was adjusted according to the following where the high level was assigned to participants scoring 24 points or more (80% or more), moderate level was assigned to participants scoring 18-23 points (60-80%), and low level was assigned to participants scoring less than 18 points (less than 60%). PPI: proton pump inhibitor

Parameter	Completely agree	Almost agree	Indifferent	Almost disagree	Completely disagree
Overuse of PPI is common at present in Saudi Arabia	88 (16.5%)	62 (11.6%)	149 (27.9%)	132 (24.7%)	103 (19.3%)
The main cause of PPI overuse is doctors' or patients' abuse of PPI	78 (14.6%)	59 (11%)	167 (31.3%)	132 (24.7%)	98 (18.4%)
The main purpose of PPI overuse is stress ulcer prophylaxis	79 (14.8%)	54 (10.1%)	181 (33.9%)	127 (23.8%)	93 (17.4%)
Overuse of PPI will cause an increase in adverse drug reactions and medical costs	72 (13.5%)	48 (9%)	158 (26.6%)	126 (23.6%)	130 (24.3%)
Necessary to carry out large-scale education on the rational use of PPI for medical staff and the public	65 (12.2%)	35 (6.6%)	126 (23.6%)	96 (18%)	212 (39.7%)
Necessary to strengthen the management of community pharmacies	56 (10.5%)	41 (7.7%)	113 (21.2%)	97 (18.2%)	227 (42.5%)

In Table 5, it is observed that a significant portion of the participants, 58.6%, reported not having used PPIs in the past year, while 21.9% confirmed using them. Additionally, when asked about the specific PPI used, a majority of participants (55.62%) responded with "I don't know", indicating a lack of awareness regarding the names of the PPIs they had used. Among those who could identify the PPI they used, omeprazole (n=68; 12.7%) was the most commonly reported. The data also sheds light on the participants' tendencies to use PPIs in response to various symptoms such as abdominal pain, nausea, vomiting, and acid reflux, showcasing varying levels of usage frequency ranging from "Never" to "Always". These findings underscore the importance of educating individuals, especially students, about the appropriate use of PPIs to ensure their optimal and safe utilization.

**Table 5:** Participants' behaviors towards PPI use* (n=534) *The other five-answer questions were graded with 1 point for always, 2 points for often, 3 for sometimes, 4 for seldom, and 5 for never. A higher score here presented a lower dependency on PPIs, corresponding to better PPI usage behavior. The total score for the four statements was 20, and scoring was adjusted according to the following where bad behavior was assigned to participants scoring 16 points or more (80% or more), moderate behavior was assigned to participants scoring 12-16 points (60-80%), and good behavior was assigned to participants scoring less than 12 points (less than 60%). PPIs: proton pump inhibitors

Parameter		No.	%
Have you used PPIs in the past year?	No	313	58.6
	Maybe	104	19.5
	Yes	117	21.9
What's the name of the PPI you used? (n=509)	I don't know	297	55.6
	Esomeprazole	24	4.49
	Omeprazole	68	12.7
	Pantoprazole	54	10.1
	Lansoprazole	26	4.87
	Rabeprazole	40	7.5
Use PPI when abdominal pain	Never	318	59.6
	Seldom	70	13.1
	Sometimes	74	13.9
	Often	43	8.1
	Always	29	5.4
Use PPI when nausea	Never	303	56.7
	Seldom	45	8.4
	Sometimes	84	15.7
	Often	48	9
	Always	54	10.1
Use PPI when vomiting	Never	314	58.8
	Seldom	49	9.2
	Sometimes	72	13.5
	Often	48	9
	Always	51	9.6
Use PPI when acid reflux	Never	288	53.9
	Seldom	30	5.6
	Sometimes	62	11.6
	Often	65	12.2
	Always	89	16.7

Table 6 in the study outlines the distribution of participants based on their attitudes towards PPI usage, categorizing them into high, moderate, and low levels. The data reveals that a significant portion of the participants, 30.9%, exhibited a high level of attitude towards PPIs, indicating a strong understanding or acceptance of their use. Meanwhile, 21% of the participants demonstrated a moderate level of attitude, suggesting a more neutral stance or some reservations about PPI usage. Surprisingly, the largest proportion of participants, 48.1%, fell into the low-level category, indicating a lack of knowledge or negative attitude towards PPIs.

**Table 6:** The attitude of participants about PPI score results* *The scoring criteria is similar to the one mentioned under Table 4. PPI: proton pump inhibitor

Attitude	Frequency	Percent
High	165	30.9
Moderate	112	21
Low	257	48.1
Total	534	100

Table 7 indicates that out of a total of 534 participants, 376 individuals, accounting for 70.4% of the sample, exhibited good behavior in relation to the use of PPIs. Additionally, 114 participants, constituting 21.3% of the total, demonstrated moderate behavior, while 44 individuals, representing 8.2% of the sample, displayed low behavior in terms of PPI usage. These findings suggest a significant proportion of participants in the study exhibit good behavior in relation to PPI use, indicating a positive trend in the understanding and appropriateness of PPI utilization among students at UQU.

**Table 7:** The behavior of participants regarding PPI score results* *The scoring criteria is similar to the one mentioned under Table 5. PPI: proton pump inhibitor

Behavior	Frequency	Percent
Good	376	70.4
Moderate	114	21.3
Bad	44	8.2
Total	534	100

A chi-squared goodness-of-fit test was conducted to determine whether the distribution of participants' behaviors regarding PPI score results (Good, Moderate, Bad) differed from an equal distribution. The results indicated a statistically significant difference between the observed and expected frequencies of behavior, χ²(2, n=534)=344.14, p<0.001. Specifically, more participants were classified as having "Good" behavior (n=376) than expected (n=178), while fewer participants than expected were classified as "Moderate" (n=114) and "Bad" (n=44).

Table 8 shows the results of the chi-squared test of independence, which was used to examine the relationship between participants' attitudes about PPIs and their sociodemographic characteristics. The test reveals a statistically significant association between participants' attitudes and their age (p=0.011), whether they belong to the medical field (p=0.0001), and their university year (p=0.007). However, the test indicates no statistically significant relationship between attitude and gender (p=0.209) or field of study (p=0.059).

**Table 8:** Relation between the attitudes of participants about PPI and their sociodemographic characteristics *P-value was considered significant if ≤0.05. The chi-squared test of independence was utilized to assess the association between participants' attitudes about PPIs and their sociodemographic characteristics. PPIs: proton pump inhibitors

		Attitude			Total (n=534)	P-value*
		High	Moderate	Low		
Gender	Female	131	214	98	443	0.209
		79.4%	83.3%	87.5%	83%	
	Male	34	43	14	91	
		20.6%	16.7%	12.5%	17%	
Age (in years)	18-30	145	196	80	421	0.011
		87.9%	76.3%	71.4%	78.8%	
	31-40	12	40	22	74	
		7.3%	15.6%	19.6%	13.9%	
	41-50	8	21	10	39	
		4.8%	8.2%	8.9%	7.3%	
Faculty of the medical field	Yes	64	138	70	272	0.0001
		38.8%	53.7%	62.5%	50.9%	
	No	101	119	42	262	
		61.2%	46.3%	37.5%	49.1%	
Field	Pharmacy	39	38	18	95	0.059
		23.6%	14.8%	16.1%	17.8%	
	Medicine	37	52	13	102	
		22.4%	20.2%	11.6%	19.1%	
	Dentistry	4	12	7	23	
		2.4%	4.7%	6.3%	4.3%	
	Nursing	16	37	15	68	
		9.7%	14.4%	13.4%	12.7%	
	Other	69	118	59	246	
		41.8%	45.9%	52.7%	46.1%	
University year	First year	27	62	25	114	0.007
		16.4%	24.1%	22.3%	21.3%	
	Second year	15	50	16	81	
		9.1%	19.5%	14.3%	15.2%	
	Third year	31	34	19	84	
		18.8%	13.2%	17%	15.7%	
	Fourth year	30	54	28	112	
		18.2%	21%	25%	21%	
	Fifth year	31	28	11	70	
		18.8%	10.9%	9.8%	13.1%	
	Sixth year	31	29	13	73	
		18.8%	11.3%	11.6%	13.7%	

Table 9 presents the results of the chi-squared test of independence, which was used to evaluate the relationship between participants' behaviors regarding PPIs and their sociodemographic characteristics. The test shows a statistically significant association between behavior and age (p=0.0001), whether the participant is in the medical field (p=0.0001), field of study (p=0.0001), and university year (p=0.049). However, the test indicates no statistically significant relationship between behavior and gender (p=0.402).

**Table 9:** Relation between the behaviors of participants about PPI and their sociodemographic characteristics *P-value was considered significant if ≤0.05. The chi-squared test of independence was utilized to assess the association between participant's behaviors regarding PPIs and their sociodemographic characteristics. PPI: proton pump inhibitor

		Behavior			Total (n=534)	P-value*
		Good	Moderate	Bad		
Gender	Female	317	90	36	443	0.402
		84.3%	78.9%	81.8%	83%	
	Male	59	24	8	91	
		15.7%	21.1%	18.2%	17%	
Age (in years)	18-30	326	61	34	421	0.0001
		86.7%	53.5%	77.3%	78.8%	
	31-40	39	29	6	74	
		10.4%	25.4%	13.6%	13.9%	
	41-50	11	24	4	39	
		2.9%	21.1%	9.1%	7.3%	
Faculty of the medical field	Yes	216	46	10	272	0.0001
		57.4%	40.4%	22.7%	50.9%	
	No	160	68	34	262	
		42.6%	59.6%	77.3%	49.1%	
Field	Pharmacy	56	26	13	95	0.0001
		14.9%	22.8%	29.5%	17.8%	
	Medicine	57	31	14	102	
		15.2%	27.2%	31.8%	19.1%	
	Dentistry	10	12	1	23	
		2.7%	10.5%	2.3%	4.3%	
	Nursing	36	25	7	68	
		9.6%	21.9%	15.9%	12.7%	
	Other	217	20	9	246	
		57.7%	17.5%	20.5%	46.1%	
University year	First year	77	26	11	114	0.049
		20.5%	22.8%	25%	21.3%	
	Second year	51	26	4	81	
		13.6%	22.8%	9.1%	15.2%	
	Third year	57	21	6	84	
		15.2%	18.4%	13.6%	15.7%	
	Fourth year	89	17	6	112	
		23.7%	14.9%	13.6%	21%	
	Fifth year	49	10	11	70	
		13%	8.8%	25%	13.1%	
	Sixth year	53	14	6	73	
		14.1%	12.3%	13.6%	13.7%	

## Discussion

PPIs are commonly prescribed medications used to treat conditions such as acid reflux, ulcers, and GERD. Inappropriate prescriptions of PPIs represent a significant financial strain both on governmental budgets and on the general population, with global costs estimated at approximately £2 billion annually. PPIs operate by reducing gastric acid production. They achieve this by targeting and inhibiting the hydrogen-potassium ATPase enzyme, commonly found in the parietal cells of the stomach. This inhibition effectively suppresses the secretion of stomach acid, thereby reducing the overall acid levels in the gastrointestinal tract [13]. PPIs increase gastric pH, which might encourage the growth of gut microflora, increase bacterial translocation, and alter various immunomodulatory and anti-inflammatory effects [14]. The long-term use of PPIs for treatment has increased as they can be obtained over the counter and outside healthcare facilities, and several studies have reported excessive use of PPIs that exceeds the number of reported cases with gastrointestinal symptoms. Thus, there is growing concern about the inappropriate use of PPIs among individuals, including students, in Saudi Arabia. A study conducted to assess the knowledge and attitudes of students towards the inappropriate use of PPIs revealed some concerning findings [13]. Many students were found to have a limited understanding of the potential risks associated with prolonged PPI use, such as increased risk of infections, nutrient deficiencies, and kidney damage. Furthermore, a significant proportion of students exhibited a casual attitude towards taking PPIs without a prescription or for non-approved indications. This lack of awareness and nonchalant attitude towards PPIs among students in Saudi Arabia highlights the need for targeted educational campaigns and interventions to promote responsible PPI use and minimize potential harm [13]. Healthcare providers, educators, and policymakers must collaborate to raise awareness about the appropriate use of PPIs and encourage students to seek professional medical advice before initiating or continuing PPI therapy. By addressing these knowledge gaps and attitudes, we can work towards ensuring the safe and effective use of PPIs among students in Saudi Arabia. Thus, we aimed in this study to assess the knowledge and attitudes towards the inappropriate use of PPIs among students and to evaluate the awareness of students regarding the potential risks associated with the inappropriate use of PPIs.

As regards participants' knowledge and awareness about the side effects of PPI, we have found that a significant proportion of participants reported experiencing common side effects such as headache, bloating, nausea, diarrhea, constipation, and abdominal pain. However, there seems to be a lack of awareness regarding more severe side effects like deterioration of kidney function, osteoporosis, and gastric cancer, as indicated by the lower percentages of affirmative responses in these categories. Moreover, the data reveals that a substantial number of participants were unaware of potential side effects such as iron deficiency anemia, mineral deficiency, and B12 deficiency. On the other hand, a study by Alshamrani et al. [15] revealed that many students believed that PPIs were harmless and could be taken without any adverse effects. Furthermore, the study showed that students had poor knowledge of the appropriate indications for PPI therapy, leading to unnecessary and potentially harmful use of these medications. Consistently, according to some studies, patient awareness of the adverse effects associated with PPIs is very low, showing that most participants were unfamiliar with any adverse effects associated with PPI use [16,17]. These studies are consistent with our results and collectively suggest a lack of awareness and understanding among students in Saudi Arabia regarding the appropriate use of PPIs, emphasizing the need for educating individuals, especially students, about the appropriate use of PPIs and the associated risks to promote safe and informed decision-making regarding their healthcare [16,17].

Regarding the attitude score of participants about PPI, we have found that a significant portion of the participants, 30.9%, exhibited a high level of attitude towards PPIs, indicating a strong understanding or acceptance of their use. Meanwhile, 21% of the participants demonstrated a moderate level of attitude, suggesting a more neutral stance or some reservations about PPI usage. Surprisingly, the largest proportion of participants, 48.1%, fell into the low-level category, indicating a lack of knowledge or negative attitude towards PPIs. On the other hand, previous studies have shown that there is a concerning attitude among students in Saudi Arabia regarding the inappropriate use of PPIs. Alshamrani et al.'s study [15] found that a significant number of students in Saudi Arabia were unaware of the potential risks associated with long-term PPI use, such as increased risk of infections, fractures, and nutrient deficiencies. Furthermore, the study revealed that many students believed that PPIs were harmless and could be taken as a preventive measure without consulting a healthcare professional.

Concerning PPI overuse among our study participants, we have found that a substantial number of participants (60.67%) reported receiving recommendations to use PPIs from sources other than healthcare professionals. In comparison, a Lebanese study found that 71.4% of the study population overused PPIs [18]. Another study found that inappropriate PPI use ranged from 40% to 81% with a mean of 63% [19]. Rotman and Bishop assessed PPI use in an ambulatory setting in the United States and found that 62.9% of PPI users had no documented gastrointestinal diagnoses/complaints or other appropriate indications [20].

As regards the behavior score of participants about PPI, we have found about 70.4% of the sample exhibited good behavior in relation to the use of PPIs. Additionally, 114 participants, constituting 21.3% of the total, demonstrated moderate behavior, while 44 individuals, representing 8.2% of the sample, displayed low behavior in terms of PPI usage. These findings suggest a significant proportion of participants in the study exhibit good behavior in relation to PPI use, indicating a positive trend in the understanding and appropriateness of PPI utilization among students at UQU. Moreover, there was a statistically significant relation to age (p=0.0001), whether the participant is in the medical field (p=0.0001), the university year (p=0.049), and the field of study (p=0.0001). It also shows a statistically insignificant relation to gender. On the other hand, sociodemographic status significantly influences sustained PPI use; as shown in the Netherlands study by Van Boxel et al. [21], low educational attainment correlates with chronic PPI utilization, emphasizing education's role in health-related attitudes and knowledge. Similarly, a nationwide Danish cohort study by Van Boxel et al. [21] linked long-term PPI use to lower income and education levels. Moreover, a study by Asdaq et al. [1] in Riyadh, Saudi Arabia, involving medical doctors, pharmacists, and nurses from diverse public and private hospitals, found that individuals with higher education, middle-aged individuals, and those with more professional experience had greater knowledge levels. Pharmacists and nurses showed lower reliance on PPIs compared to doctors. The research also revealed a positive correlation between healthcare professionals' attitudes, knowledge, and behaviors regarding PPI usage in Saudi Arabia. Another study by Alasmari et al. [22] focused on physicians, pharmacists, and clinical pharmacists in Saudi Arabia, revealing significant disparities in PPI awareness, with more reporting poor knowledge.

Limitations

While this study offers valuable insights into the knowledge and attitudes of UQU students regarding the inappropriate use of PPIs, several limitations should be considered.

First, the study's sample, though statistically adequate at 534 participants, is limited to a single institution in Makkah, which may restrict the generalizability of the findings to other universities or regions with different educational and healthcare environments. As such, the results may not fully represent the broader student population of Saudi Arabia.

Second, the reliance on self-reported data introduces the possibility of reporting bias, where participants may overestimate their knowledge or provide socially desirable responses regarding their PPI usage behaviors. This limitation could affect the accuracy of the data collected and the conclusions drawn from the study.

Additionally, the use of a self-designed questionnaire, while refined through expert feedback and pilot testing, may not capture all nuances related to PPI knowledge and attitudes. Furthermore, while the sample size meets statistical requirements, constraints related to research team manpower, time, and available funding may have limited the ability to recruit a larger sample or conduct more detailed subgroup analyses, which could have provided deeper insights, particularly when comparing healthcare and non-healthcare students.

Recommendations

To effectively address the knowledge gaps identified in our study, we recommend implementing targeted educational workshops integrated into health sciences curricula, ensuring all healthcare students understand the appropriate use, indications, and potential risks of PPIs. Accessible educational materials, such as brochures and online resources, should be developed to enhance student understanding. Establishing peer-led education programs can further engage students and facilitate knowledge transfer. Collaborating with local healthcare professionals to host seminars will provide practical insights into best prescribing practices. Additionally, training healthcare providers on PPI prescription guidelines and monitoring prescriptions through pharmacists can promote responsible usage. A feedback mechanism should be established to assess changes in knowledge and attitudes following these interventions. Lastly, public awareness campaigns aimed at both healthcare professionals and the broader community will raise awareness about the risks of inappropriate PPI use, encouraging informed decision-making. By implementing these strategies, we aim to improve understanding and promote safe PPI usage among students and the wider community in Makkah.

## Conclusions

While the respondents showed good behavior levels, there is a lack of good attitude surrounding the use of PPI medications. It is evident that there is a pressing need for educational interventions to be implemented in order to improve the understanding of the appropriate indications and potential risks associated with PPIs. By addressing the gaps in knowledge and attitudes towards these medications, healthcare professionals can work towards promoting safe and effective use among students in Makkah.

## Appendices

Questionnaire

Section A: Demographics

**Table 10:** Section A

No.	Question	Answer
1	Gender	□ Male
		□ Female
2	Age (in years)	□ 18-30
		□ 31-40
		□ 41-50
3	Faculty of the medical field	□ Yes
		□ No
	If yes, field	□ Medicine
		□ Pharmacy
		□ Nursing
		□ Dentistry
		□ Other
4	Students' ID	
5	University year	□ First year
		□ Second year
		□ Third year
		□ Fourth year
		□ Fifth year
		□ Sixth year

Section B: Knowledge of Participants of Proton Pump Inhibitors (PPIs)

Participants who used PPIs can choose more than one indication at the same time.

**Table 11:** Section B PPI: proton pump inhibitor

No.	Question	Answer
1	Have you ever used PPI before?	□ Yes, by medical consultation
		□ Yes, without medical consultation
		□ I have never used it
2	If yes, the reason for using PPI?	□ Peptic ulcer
		□ Gastroesophageal reflux disease
		□ Heartburn
		□ *Helicobacter pylori* infection
		□ Esophagitis
		□ Hiatal hernia
		□ Asthma
		□ Hoarseness
		□ Gastritis
		□ Gastroenteritis
		□ Sore throat
		□ After bariatric surgery
		□ I have not used it before

Section C: Duration of Using the Drug and Course Completion Among Participants Who Used PPIs

**Table 12:** Section C PPI: proton pump inhibitor

No.	Question	Answer
1	Who recommended using PPI?	□ Physician
		□ Pharmacist
		□ Friend
		□ Others
2	Duration of using PPI if used	□ As needed
		□ Regularly more than four weeks
		□ Regularly less than four weeks
		□ Not regularly
		□ Used the medication but did not complete the recommended duration
		□ I have not used it before
3	Appropriate time to take PPI	□ Before meal
		□ After meal
		□ With meal
		□ I have not used it before
4	Completed the course, if used PPI	□ Yes
		□ No
		□ Maybe
		□ Other
	If no, why?	□ Due to side effects
		□ Fear of side effects
		□ Not affordable, not available
		□ Symptoms disappeared, no longer needed
		□ Advice from a friend or relative (in the medical field)
		□ Advice from a friend or relative (outside the medical field)
		□ Missed
		□ I have not used it before

Section D: Participants' Use and Knowledge of PPIs' Side Effects

**Table 13:** Section D ^¶^Hypothesized yet unproven side effects that are linked to PPIs. PPIs: proton pump inhibitors

No.	Question	Answer
1	Headache	□ Yes
		□ No
2	Bloating	□ Yes
		□ No
3	Nausea	□ Yes
		□ No
4	Diarrhea	□ Yes
		□ No
5	Constipation	□ Yes
		□ No
6	Deterioration of kidney function	□ Yes
		□ No
7	Abdominal pain	□ Yes
		□ No
8	Osteoporosis	□ Yes
		□ No
9	Iron deficiency anemia	□ Yes
		□ No
10	Mineral deficiency	□ Yes
		□ No
11	Increased risk of gastroenteritis	□ Yes
		□ No
12	Pneumonia	□ Yes
		□ No
13	*Clostridium difficile* infection	□ Yes
		□ No
14	B12 deficiency	□ Yes
		□ No
15	Gastric cancer^¶^	□ Yes
		□ No
16	I haven't used it before	□ Yes
		□ No

Section E: The Attitude of Respondents Regarding PPI Use

**Table 14:** Section E PPI: proton pump inhibitor

No.	Question	Answer
1	Overuse of PPI is common at present in Saudi Arabia	□ Completely agree
		□ Almost agree
		□ Indifferent
		□ Almost disagree
		□ Completely disagree
2	The main cause of PPI overuse is doctors' or patients' abuse of PPI	□ Completely agree
		□ Almost agree
		□ Indifferent
		□ Almost disagree
		□ Completely disagree
3	The main purpose of PPI overuse is stress ulcer prophylaxis	□ Completely agree
		□ Almost agree
		□ Indifferent
		□ Almost disagree
		□ Completely disagree
4	Overuse of PPI will cause an increase in adverse drug reactions and medical costs	□ Completely agree
		□ Almost agree
		□ Indifferent
		□ Almost disagree
		□ Completely disagree
5	Necessary to carry out large-scale education on the rational use of PPI for medical staff and the public	□ Completely agree
		□ Almost agree
		□ Indifferent
		□ Almost disagree
		□ Completely disagree
6	Necessary to strengthen the management of community pharmacies	□ Completely agree
		□ Almost agree
		□ Indifferent
		□ Almost disagree
		□ Completely disagree

The next six questions, which were related to attitude, were regulated based on the 5-point Likert scale with the scores being as follows [18]: 5 for "completely agree", 4 for "almost agree", 3 for "indifferent", 2 for "almost disagree", and 1 for "completely disagree". A higher score in the first two categories represented better awareness of PPIs or a more positive attitude.

Section F: The Behavior of Respondents Towards PPI Use

**Table 15:** Section F PPI: proton pump inhibitor

No.	Question	Answer
1	Have you used PPIs in the past year?	□ Yes
		□ No
		□ Maybe
If you have used PPI, then the next six questions will be answered.		
2	What's the name of the PPI you used?	□ Omeprazole
		□ Pantoprazole
		□ Lansoprazole
		□ Rabeprazole
		□ Esomeprazole
		□ I have not used it last year
3	Use PPI when abdominal pain	□ Always
		□ Often
		□ Sometimes
		□ Seldom
		□ Never
4	Use PPI when nausea	□ Always
		□ Often
		□ Sometimes
		□ Seldom
		□ Never
5	Use PPI when vomiting	□ Always
		□ Often
		□ Sometimes
		□ Seldom
		□ Never
6	Use PPI when acid reflux	□ Always
		□ Often
		□ Sometimes
		□ Seldom
		□ Never
